# Algorithm with Patterned Singular Value Approach for Highly Reliable Autonomous Star Identification

**DOI:** 10.3390/s20020374

**Published:** 2020-01-09

**Authors:** Kiduck Kim, Hyochoong Bang

**Affiliations:** Department of Aerospace Engineering, Korea Advanced Institute of Science and Technology, 291 Daehak-ro, Yuseong-gu, Daejeon 34141, Korea; kdkim330@kaist.ac.kr

**Keywords:** agile satellite, star tracker, star identification, star pattern recognition, singular value, pattern matching

## Abstract

In the work reported in this paper, a lost-in-space star pattern identification algorithm for agile spacecraft was studied. Generally, the operation of a star tracker is known to exhibit serious degradation or even failure during fast attitude maneuvers. While tracking methods are widely used solutions to handle the dynamic conditions, they require prior information about the initial orientation. Therefore, the tracking methods may not be adequate for autonomy of attitude and control systems. In this paper a novel autonomous identification method for dynamic conditions is proposed. Additional constraints are taken into account that can significantly decrease the number of stars imaged and the centroid accuracy. A strategy combining two existing classes for star pattern identification is proposed. The new approach is intended to provide a unique way to determine the identity of stars that promises robustness against noise and rapid identification. Moreover, representative algorithms implemented in actual space applications were utilized as counterparts to analyze the performance of the proposed method in various scenarios. Numerical simulations show that the proposed method is not only highly robust against positional noise and false stars, but also guarantees fast run-time, which is appropriate for high-speed applications.

## 1. Introduction

Recently, there has been an increasing demand for spacecraft with fast attitude maneuverability. Such high maneuverability is called agility, and agile spacecraft have the advantage of being able to image more ground targets than a non-agile satellite could in the same time. Because mobility is applicable to synthetic aperture radar (SAR) satellites, which track moving ground targets, it is being developed and operated for a variety of purposes, including commercial and military satellites [1]. Some examples include Pleiades (France) [2,3,4], COSMO-SkyMed 2nd generation (Italy), and the Worldview satellites (USA), and the interest in agile satellites as future space platforms is steadily growing. These satellites require 1–10 deg/s range of fast slew maneuvers [5]. In these high-performance satellites, fine attitude determination is a very important capability related to rapid maneuverability. There are many types of attitude-sensing devices such as star trackers, magnetometers, sun sensors, and horizon sensors. However, star trackers are the most frequently used in modern attitude control systems. This is because they can provide high-fidelity three-axis attitude information within a few arc-seconds of accuracy. Therefore, a star tracker is necessary in agile satellites to provide improved missions with broader range. However, the operation of star trackers is known to be poor during fast maneuvers [6,7,8].

A star tracker requires very sophisticated hardware and software technologies for high-rate applications. The greatest difference from making an image of a stationary star is that the stars imaged are elongated across neighboring pixels due to motion of the imagery sensor during the exposure time. The result is not a simple point source as in the static condition. This is a significantly challenging condition for the sensors, which causes a predicament in their operation. The primary difficulty is to reduce the signal-to-noise ratio (SNR), one of the main performance parameters of star trackers. When the elongation length is increased, the peak intensity of a star is spread out across several pixels. This not only prevents detection of dimmer stars, but also degrades centroid accuracy. For this reason, many researchers have investigated the use of star tracker systems for fast slewing conditions. In [8,9,10], researchers analyzed the effect of dynamic conditions and tried to solve the related problems by adjusting the optical design parameters. Other researchers proposed adaptive image processing techniques [11,12], and an improved centroid algorithm [13] to achieve identical performance even at a high rate of sensor operation. In addition, methods for tracking stars across sequential images were proposed [6,7] utilizing prior information.

The goal of this work was to create an autonomous star identification algorithm for lost-in-space (LIS) scenarios to address the high-rate application problem of star trackers. In spite of many previous studies to accommodate dynamic conditions, there have been few studies on the aspects of the identification process. Star identification in LIS mode is essential for star trackers, and could thus serve as a direct measure of its performance [14,15,16]. Of course, some recent literature has focused on fast identification of stars for agile spacecraft or for fast recovery after an abrupt power outage [17,18]. However, they did not focus on the dynamic conditions that change with rapid attitude maneuvers. Thus, the aim of this study was to develop a fast and highly reliable identification algorithm that could broaden the autonomy of the attitude control system of agile spacecraft. While the focus of most previous studies was on considering as many constraints as possible in the autonomous identification problem, consideration of only selected constraints critical during rapid angular rates has not been fully investigated yet. A new method for identification is considered herein by which such dynamic conditions can be considered to fill this gap.

There have been two areas of major progress. First, the dynamic condition is now being taken into account. As explained in [19], agile satellites with high angular maneuverability could significantly decrease the number of stars to be imaged, compared to a static condition. This additional constraint makes it very difficult to provide an algorithm able to promise robustness under this condition. For this reason, an innovative three-step matching method is proposed. Second, to evaluate the robustness and time effectiveness of the proposed algorithm, representative algorithms, including those actually implemented for space applications (e.g., pyramid [20] and grid [21] as well as modified grid [22] algorithms) and widely used for performance comparison, were benchmarked.

In the remainder of this paper, Section 2 summarizes previous star-identification algorithms and explains a pattern-extraction method. Section 3 explains matching schemes of the proposed method. Section 4 provides analysis of the dynamic conditions that should be considered in database generation. Section 5 presents exhaustive numerical simulation results and discussion to support the proposed method. Section 6 includes the conclusions and final remarks.

## 2. Innovative Pattern Extraction

### 2.1. Previous Identification Algorithm

Many algorithms for autonomous star identification have appeared during the last four decades (e.g., Junkins et al. [23]). The strategies adopted in those algorithms can be categorized into two classes in accordance with the features they use to identify stars. Algorithms in the first class conjugate invariant properties from coordinate transformation to find a unique identity for an imaged star. These algorithms compare angular distance or singular values between adjacent stars with those of catalogued stars to determine if an imaged star is in the star database. This class of algorithm is a classical and mature method in the field and includes what is called the triangle algorithms, which include the oldest algorithms in this category [23,24]. However, these methods suffer from difficulty matching stars when there is image noise because only three invariants from a triangle are not sufficient to find a star with a unique match.

In other research, scholars have proposed ways to address such drawbacks (e.g., group match [25], pyramid [20], and polygon-based [26] methods). The aim of those algorithms was to enhance a simple feature by employing a higher number of invariants to decrease incorrect matching among a large number of candidates. M. Kolomenkin et al. [16] proposed a geometric voting algorithm utilizing two voting procedures to guarantee more robustness against positional and false star noise. More recently, algorithms using singular value decomposition (SVD) were proposed in this category instead of angular distance [27,28,29]. It was shown that the singular value from measured unit vectors is also preserved from coordinate transformation and this allows merging of the identification process with the attitude estimation process.

In spite of many alterations, algorithms of the first class are known to be unreliable in the presence of image noise, and require massive memory usage for database construction relative to the second class of algorithms [30]. For the last two decades, star identification algorithms have mostly been of the second class of algorithms, whereby surrounding stars are utilized as a pattern. The grid algorithm is the most representative one in this category (as proposed by Padgett et al. [21]). The Cartesian coordinates of stars are discretized in the defined grid pattern, and surrounding stars are treated as a binary marker of the pattern. If a star is located in a grid cell, then its value is regarded as 1; otherwise, it is 0. As a result, a binary pattern is constructed that can be compared with that of the database to find the closest similarity.

Indeed, this is an innovative algorithm relative to algorithms of the first class, and is obviously robust against image noise. Moreover, it also has a shorter identification time due to generation of a lookup table. The advantages of the grid algorithm have fostered new possibilities for pattern-matching techniques for star identification. Many associated proposals have been made to the grid algorithm to improve its performance. Most researchers have focused their attention on the problem of choosing the closest neighboring star, which should be selected to define a grid in the Cartesian coordinate system. The polar coordinate system was introduced in such algorithms to avoid incorrect selection of the closest neighboring star by making patterns based on radial directions [22,31,32,33,34]. These radial-based patterns have been used in state-of-the-art algorithms, which are known to provide more robustness than is possible with the original grid algorithm.

Under dynamic conditions that not only require fast identification, but also tend to degrade the centroid accuracy, algorithms of the second-class provide more suitable solutions. Because all the previous methods utilize the stars surrounding the target star as patterns, better performance is achieved by using more stars to make a complex pattern. Thus, the majority of the studies presented algorithms that even use quite dark stars. However, the usage of dim stars is constrained because of the large errors in their high angular rates. This is a major problem that degrades the performance of the existing algorithms.

The contribution of this paper includes the successful fusion of the two previous classes for star pattern identification. To the best of our knowledge, there have been no studies combining the two classes before, and the main objective was to construct efficient patterns using singular values to complement the drawbacks of each class of algorithms. Regarding the aspects of algorithms of the first class, adopting a pattern-matching scheme allows a singular value to be more committed to robustness of the algorithm than when it is directly compared with those of a database. In addition, the use of very complicated features avoids the exhaustive search process typical with the usual algorithms in the first class. These algorithms search major combinations of geometry among neighboring stars to improve their identification rates. This leads to longer running time and requires a lot of memory usage, which are inherent disadvantages of algorithms.

Regarding the aspects of algorithms of the second class, the issue of the closest neighboring star disappears, as in the improvements in other recent algorithms. An axis definition is no longer required in the proposed pattern because singular values were used instead of imaged stars. This paper provides more innovative patterns which promise enhanced robustness in dynamic conditions. In addition to a fundamental discretization process, the singular values are complicated by constructing a grid on invariant space. The discretized singular values provide a pattern with one more degree of freedom (replacing the simple binary feature (not just 0 or 1) to indicate the existence of stars). This further complicates the pattern and makes it possible to identify stars using a reduced number of stars. Details of the proposed pattern are discussed in the next section.

### 2.2. Introduction of Singular Values

This section is intended to provide brief information to readers who may not be familiar with the invariant properties of singular values. The singular value decomposition method has been widely used in applications involving signal processing, image processing, and attitude estimators by providing meaningful information about associated matrices. It is known to be one of the most robust algorithms in numerical algebra [27]. Readers can refer to the analytical robustness of singular values with respect to error factors on star images, in particular [29].

**Figure 1:**
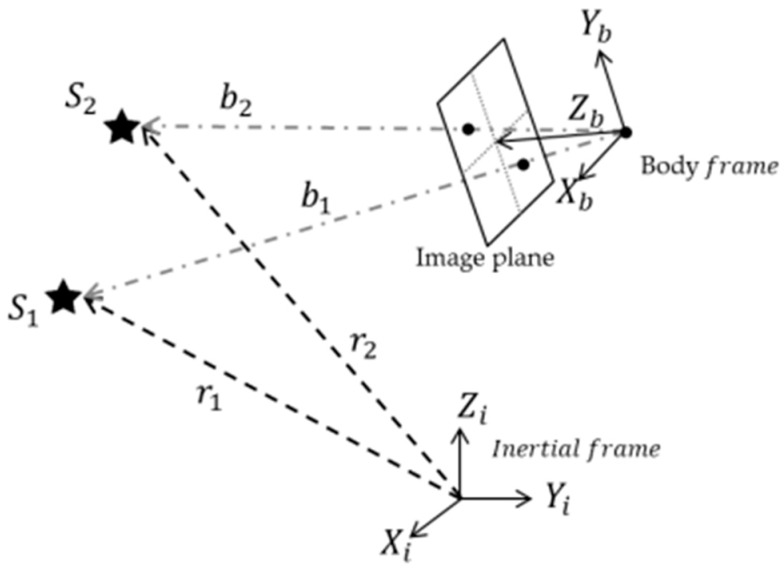
Vector measurements model of a star sensor.

The first class of algorithms was developed to utilize angular distance and singular values because they are invariant under coordinate transformation. The vector measurement of a star tracker is the rotation of the star reference-unit vector from inertial to body frame, as denoted in Figure 1. Let $r_{i}$ be the $i_{th}$ star reference-unit vector expressed in the inertial frame, and $b_{i}$ is the corresponding vector in the body frame.

Their relationship is generally given as

(1) $$b_{i}=C\cdot r_{i},$$

where *C* is the direction cosine matrix (DCM) that rotates an arbitrary vector from the inertial frame to the body frame. This rotation matrix is a unitary matrix satisfying.

(2) $$CC^{T}=C^{T}C=I_{3\times 3},$$

(3) $$C^{-1}=C^{T},$$

where *I* is an identity matrix in three dimensions. For N stars in the image plane, one can rotate 3 × *N* column vector matrices R in the inertial frame to 3 × *N* column vector matrices B in the body frame. The subscripts of R and B represent the size of each matrix.

(4) $$B_{3\times N}=C\cdot R_{3\times N}.$$

The column vector matrices R and B are given with the unit vector of *N* stars.

(5) $$R_{3\times N}=\left[\begin{array}{cccc} r_{1} & r_{2} & \cdots & r_{N} \end{array}\right],$$

(6) $$B_{3\times N}=\left[\begin{array}{cccc} b_{1} & b_{2} & \cdots & b_{N} \end{array}\right].$$

Then, R and B can be factorized by singular value decomposition.

(7) $$R_{3\times N}=P_{r}D_{r}Q_{r}^{T}=\sum_{i=1}^{3}p_{r,i}\sigma_{r,i}q_{r,i}^{T},$$

(8) $$B_{3\times N}=P_{b}D_{b}Q_{b}^{T}=\sum_{i=1}^{3}p_{b,i}\sigma_{b,i}q_{b,i}^{T},$$

where $P_{r}$ and $P_{b}$ are 3×3 orthogonal matrices of left singular vectors, which are the same as the normalized eigenvectors of $RR^{T}$ and $BB^{T}$, respectively. Here, $D_{r}$ and $D_{b}$ are 3×N diagonal matrices with singular values $\sigma_{r,i}$ and $\sigma_{b,i}$ of R and B. The terms $Q_{r}$ and $Q_{b}$ denote N×N orthogonal matrices of right singular vectors, which are the same as the normalized eigenvectors of $R^{T}R$ and $B^{T}B$, respectively. Because the unit vectors are three-dimensional (3-D) with components in x, y, and z directions, the rank of the two matrices R and B is 3 for N≥3 star numbers. Thus, there are always three non-zero singular values for any number of N≥3 stars.

When we post-multiply $B_{3\times N}^{T}$ over Equation (4), we obtain Equation (9), which is the form of the similarity transformation because of the unitary property of direction cosine matrix C in Equation (3).

(9) $$B_{3\times N}B_{3\times N}^{T}=C\cdot R_{3\times N}\;R_{3\times N}^{T}{}_{3\times N}C^{-1}.$$

The formulation in Equation (9) is a similarity transformation between two 3×3 real and squared symmetric matrices $R_{3\times N}R_{3\times N}^{T}$ and $B_{3\times N}B_{3\times N}^{T}$. Substituting Equations (7) and (8) into Equation (9) leads to

(10) $$C\;R_{3\times N}R_{3\times N}^{T}\;C^{-1}=C\;P_{r}D_{r}Q_{r}^{T}Q_{r}D_{r}^{T}P_{r}^{T}C^{-1}=CP_{r}{\tilde{S}}_{r}P_{r}^{T}C^{-1},$$

(11) $$B_{3\times N}B_{3\times N}^{T}=P_{b}D_{b}Q_{b}^{T}Q_{b}D_{b}^{T}P_{b}^{T}=P_{b}{\tilde{S}}_{b}P_{b}^{T}.$$

Then, Equation (12) can be expressed from Equations (10) and (11) as

(12) $$P_{b}\;{\tilde{S}}_{b}\;P_{b}^{T}=C\;P_{r}{\tilde{S}}_{r}P_{r}^{T}C^{-1}.$$

Here, ${\tilde{S}}_{r}$ and ${\tilde{S}}_{b}$ are 3×3 diagonal matrices with eigenvalues $\sigma_{r,i}^{2}$ and $\sigma_{b,i}^{2}\;(i=1,\;2,\;3)$, as given by

(13) $${\tilde{S}}_{r}=D_{r}D_{r}^{T},$$

(14) $${\tilde{S}}_{b}=D_{b}D_{b}^{T}.$$

Because the eigenvalues are preserved from the similarity transformation in Equation (9), we obtain the relationship between $\sigma_{r,i}$ and $\sigma_{b,i}$

(15) $${\tilde{S}}_{r}={\tilde{S}}_{b},$$

or

(16) $$\begin{array}{ccc} \sigma_{r,1}^{2}=\sigma_{b,1}^{2},\; & \sigma_{r,2}^{2}=\sigma_{b,2}^{2}, & \sigma_{r,3}^{2}=\sigma_{b,3}^{2} \end{array}.$$

This implies that R and B apparently possess the same singular values with the change of direction cosine matrix caused by coordinate transformation.

(17) $$\begin{array}{ccc} \sigma_{r,1}=\sigma_{b,1},\; & \sigma_{r,2}=\sigma_{b,2}, & \sigma_{r,3}=\sigma_{b,3} \end{array}.$$

### 2.3. Extract Singular Values

**Figure 2:**
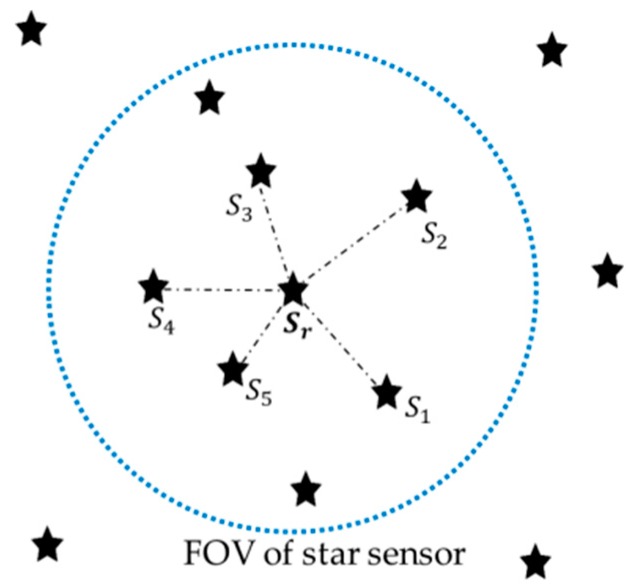
Illustration of the selected reference star and nearest five stars.

The proposed algorithm generates a pattern with a specific number of imaged stars. It requires six stars for the pattern, one of which is the reference star that we want to identify; the other five stars are used to construct a unique pattern. Prior to generating a pattern, the singular values should be computed using the imaged stars. To avoid further ambiguity, it should be noted that brightness information is completely excluded from this method. Then, the five nearest stars will be selected because, without brightness information, the only way remaining is to make use of the distance in the image plane. The illustration of the 6-star pattern is shown in Figure 2. The reference star is $S_{r}$ and the nearest five stars can be distinguished by their subscripts: $S_{1}$, $S_{2}$, $S_{3}$, $S_{4}$, and $S_{5}$ in the figure.

The next step is to make subsets by clustering four of the nearest five stars. For uniqueness of the pattern, the important thing here is that the reference star $S_{r}$ must be included in each set to lead to a distinct pattern between $S_{r}$ and other reference stars. According to this rule, only five sets are feasible and every set contains five stars. The five sets can be expressed as

(18) $$Set_{1}=\left\{\begin{array}{cccc} S_{r}; & S_{1}, & S_{2}, & \begin{array}{cc} S_{3}, & S_{4} \end{array} \end{array}\right\},$$

(19) $$Set_{2}=\left\{\begin{array}{cccc} S_{r}; & S_{1}, & S_{2}, & \begin{array}{cc} S_{3}, & S_{5} \end{array} \end{array}\right\},$$

(20) $$Set_{3}=\left\{\begin{array}{cccc} S_{r}; & S_{1}, & S_{2}, & \begin{array}{cc} S_{4}, & S_{5} \end{array} \end{array}\right\},$$

(21) $$Set_{4}=\left\{\begin{array}{cccc} S_{r}; & S_{1}, & S_{3}, & \begin{array}{cc} S_{4}, & S_{5} \end{array} \end{array}\right\},$$

(22) $$Set_{5}=\left\{\begin{array}{cccc} S_{r}; & S_{2}, & S_{3}, & \begin{array}{cc} S_{4}, & S_{5} \end{array} \end{array}\right\}.$$

Refer to $set_{1}$: This set is also divided into subsets to employ additional singular values. The stars in this set are arranged in subsets that have at least three stars. In this way, the proposed method uses the cascade concept. If we want to make subsets that contain five stars, the reference star $S_{r}$ is always selected and four stars are picked out from remaining stars (that is, $S_{1}$, $S_{2}$, $S_{3}$, and $S_{4}$ in the case of $Set_{1}$). Similarly, the number of stars in the subsets is steadily decreased to four and three, and then the associated number of stars to be picked out (except for the reference star) is also three and two, respectively. This is the same as a combination problem using four, so that only one subset of five stars (=C44), four subsets of four stars (=C34), and six subsets of three stars (=C24) exist. The singular values can be evaluated from the measured unit vectors in each of the eleven subsets according to the procedures in the previous section. An identical process is applied from $Set_{2}$ to $Set_{5}$ to compute singular values, so that every set contains eleven subsets as a result. Before we move on to the next section to describe how to construct discretized patterns, an example is given to clarify the description and explain an idea for the initial match by which to reduce candidates efficiently.

**Figure 3:**
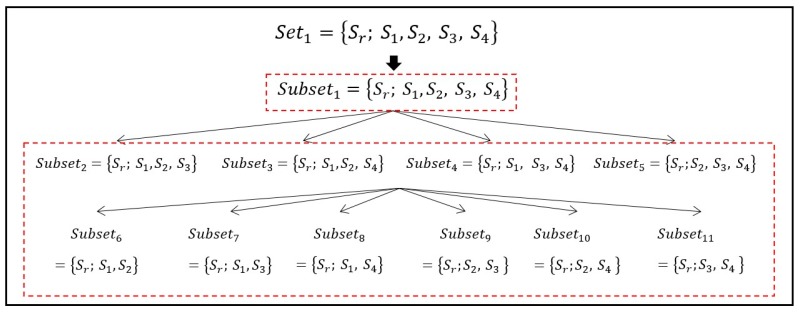
Example of cascade of 11 subsets in the case of $Set_{1}$.

Figure 3 shows an example of constructed subsets for one set of a reference star (denoted as $subset_{i}$), where i represents indices of the subset (i=1, 2,⋯11). As one can see in the figure, the subsets are divided into two parts by the boxes. Here, $subset_{1}$ (in the first box) is used for the initial match. Then, database stars that are not similar to singular values from $subset_{1}$ are filtered out in the initial match, so that they are not considered as candidates. The principal advantage of this idea is that there is no indexing issue. As long as the same five stars are used, identical singular values can be obtained regardless of the selection order. We only select five stars and no other information is needed for the initial match. With the help of the initial match, the algorithm has the potential to save time needed for identification and to provide more reliable performance by eliminating candidates from the database. This reduces the number of stars that need to be compared.

### 2.4. Discretization and Pattern Generation

**Figure 4:**
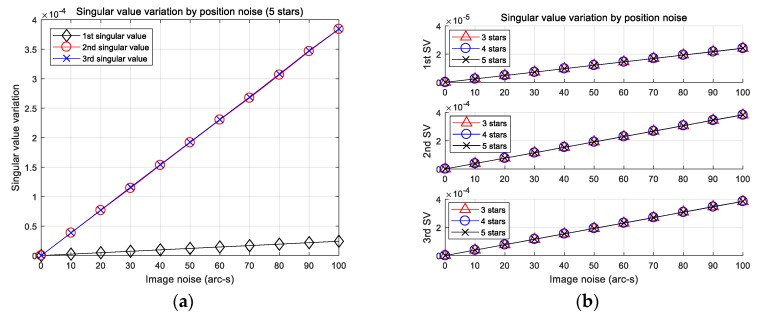
(**a**) Average error of each singular value of subsets of five stars; (**b**) comparison of average error of subsets of three, four, and five stars.

The discretization step size is the first consideration in the algorithm, which may be directly related to its performance. It should be determined considering that it be not only large enough to mitigate the position error, but also small enough not to lose its pattern complexity. The conventional method use sensor coordinates, so it is possible to adjust easily the step size through pixel units. On the other hand, our study does not apply in this case because singular values are used. Therefore, it was necessary to determine how the singular values are changed by position error. To analyze such variation, the position errors were introduced as x and y coordinates (generated by random Gaussian noise). The 1σ value of standard deviation was increased from 0 to 100 arc-seconds, and 10,000 simulations were performed at each noise level. The simulations are carried out by assuming the 12.09 deg field of view. For other specifications of the sensor, readers are referred to the dynamic environment analysis section. Figure 4 shows average error against position error.

Note that the three singular values computed are distinguished by their norm (i.e., the largest singular value represented as the first singular value). The results of the left figure indicate that the first singular value has been changed less than the other two, and that the changes of the other two values are almost identical. These results imply that the step size has to be determined differently between the first and other singular values by taking into account the amount of error. Moreover, the right-side plots indicate that the average errors of the singular values are very similar despite the difference in the number of stars in the subsets. Based on the results, knowing the step size is not necessary to consider the number of stars in a subset. In this paper, it was roughly decided to use values slightly larger than the error values at 100 arc-seconds to make the pattern less susceptible to position error. The parameters defined and the distribution ranges of each subset are listed in Table 1.

After the step size was determined, three singular values of each subset were turned into a grid pattern using the discretization process. First, a cell index is defined with the second and third singular values because they have similar distribution ranges as well as the same step size. To obtain the relationship between singular values and cell index, we can make them integers using the following equations.

(23) $${\bar{s}}_{x}=floor\left(\frac{sv_{2}}{gs_{2}}\right)+1,$$

(24) $${\bar{s}}_{y}=floor\left(\frac{sv_{3}}{gs_{3}}\right)+1.$$

Here, ${\bar{s}}_{x}$ and ${\bar{s}}_{y}$ are discretized integer numbers of the second and third singular values, and ${gs}_{k}$ and $sv_{k}$ are the associated predefined step size and singular values, respectively. Here, the subscript k denotes the index of singular values such that k=2, 3. The function *floor(x)* rounds *x* to the nearest integer toward negative infinity. Then, the cell index of the selected subsets can be represented as

(25) $${\bar{c}}_{xy}\left(\begin{array}{cc} {\bar{s}}_{x}, & {\bar{s}}_{y} \end{array}\right)=\left({\bar{s}}_{y}-1\right)\times rs_{x}+{\bar{s}}_{x},$$

(26) $$rs_{x}=floor\left(\frac{\mathrm{Distribution}\;\mathrm{range}}{gs_{2}}\right),$$

where ${\bar{c}}_{xy}$ is a cell index for each subset and $rs_{x}$ is an *x*-axis resolution of a grid pattern that can be expressed with a function of the step size and distribution range of the second singular value. For example, $rs_{x}$ becomes 100 $\left(=0.15/\left(15\times 10^{-4}\right)\right)$ in the case of subsets of five stars.

Finally, the first singular value is discretized to constitute the values in the cell index. This is a major difference that allows the proposed method to formulate well-defined patterns. The value in the cell index ranges from zero to resolution of the first singular value (i.e., 80 $\left(=\left(2.238-2.23\right)/\left(1\times 10^{-4}\right)\right)$ for subsets of five stars, whereas it is a binary value (0 or 1) in conventional algorithms according to the existence of a star (or not).

The discretized first singular value ${\bar{s}}_{1}$ is given by

(27) $${\bar{s}}_{1}=floor\left(\frac{sv_{1}}{gs_{1}}\right)+1.$$

Then, we can obtain the pattern for each subset denoted as

(28) $$pat_{subset}=\left(\begin{array}{cc} {\bar{c}}_{xy}, & {\bar{s}}_{1} \end{array}\right).$$

## 3. Matching Scheme for Identification

**Figure 5:**
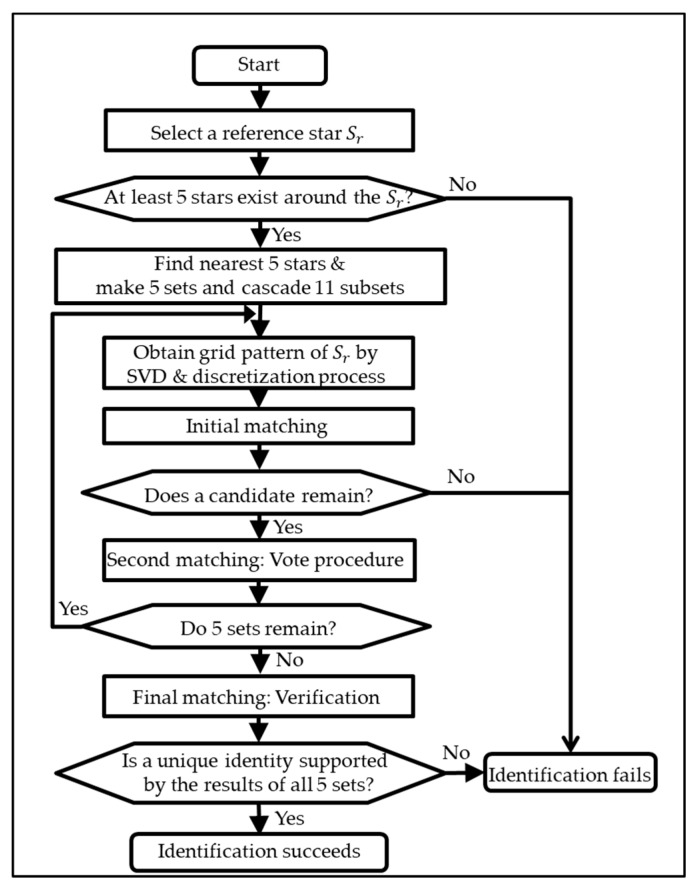
Flow chart of the single identification procedure.

Once the patterns were configured, imaged stars could be identified using a sequence of three matching schemes. Figure 5 shows a flow chart of a single identification process in the proposed algorithm. It starts from the initial matching stage to reduce the candidates in the database. As mentioned earlier, the pattern constructed from the subset of five stars was used for this purpose (see Figure 3). The candidates remaining after the initial match, only then were compared with the reference star. In this second stage, one of the candidates was selected as the reference star of Set 1 by a voting procedure. The voting procedure used 10 patterns from subsets of three and four stars to find the most similar patterns between the imaged and catalogued stars. Both the initial match and voting procedures were repeated for every five sets of the reference star (in Equations (18)–(22)).

Of course, the reference star might be misidentified as other stars in the catalogue in spite of the two matching schemes. Because there might exist similar patterns among catalogue stars, and because image noise could perturb a unit vector of an imaged star, the final step was to verify the results to prevent such spurious matching. By this process, a result was adopted from each of the five sets of the reference star. If they came to a single identity, that identity was considered the final identification result of the reference star. Detailed explanation and examples are given in the following sections.

### 3.1. Initial Matching Step

The initial matching step requires a threshold to indicate differences in the patterns of the subsets of the five stars. In this paper, this threshold was set to 1 in consideration of the ambiguity caused by position error and other factors. This means that only stars in the database with a difference of <1 for the pattern were considered as candidates. Table 2 shows a brief example of the initial matching to show clearly the threshold effect.

It is noteworthy to mention that this approach achieves effective usage of the database by limiting the search area without the look-up tables used in conventional pattern-matching schemes. This section describes only the threshold value, and how it works in the initial matching. For how much the search area is reduced, readers are referred to a quantitative discussion in the simulation study section.

### 3.2. Voting Process for the Remaining Candidates

In the second matching, the candidates remaining after the initial match are compared with the imaged star to select one candidate for each set of reference stars. The voting is done to investigate the similarity among the ten grid patterns. The similarity of patterns is then replaced by the number of votes in which a maximum similarity score is 10 (two patterns are perfectly matched). If a candidate gets a voting score satisfying a defined threshold value, it is recorded for the next (verification) step; otherwise, it is discarded. One thing to be noted here is that this algorithm was able to provide a consistent threshold for an entire sky scene because it utilized a fixed number of patterns. The pattern numbers of other matching algorithms change according to the surrounding stars, so they are likely to show incoherent performance in terms of the number of stars changed by optical parameters or by orientation in the sky.

**Figure 6:**
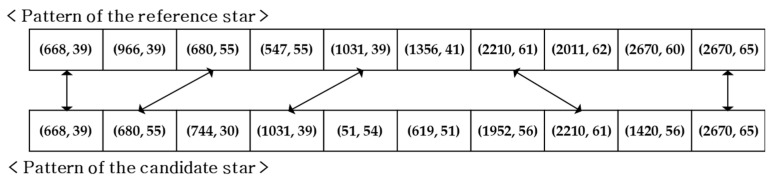
Example of scoring in the voting procedures with 10 singular value grid patterns.

In this paper, the threshold was defined as 7 to attain high reliability under the dynamic condition. A procedural example is represented in Figure 6. In this case, five identical patterns were found in the comparison of the patterns of the reference and candidate star. Therefore, in this case of only five matches, the candidate would be discarded and the algorithm would go through the other remaining candidates to check their vote scores.

### 3.3. Final Verification of the Identification

The results of two matching schemes were verified in this final step to enhance reliable performance of the algorithm. It is an intuitive process that the unique identity of the imaged star will be finalized only when it is supported by the results of the five sets. Table 3 shows the voting results of each set of one reference star. In the second process, the identifying match may be recorded as −1 if a candidate does not meet the threshold, or if more than one candidate satisfies the threshold. The result of $set_{4}$ in the table is a former case, and of course it is excluded from the verification step. One can also see that the result of $set_{2}$ is matched erroneously even though it satisfies the defined threshold with seven votes. However, it is not supported by the other results of $set_{1}$, $set_{3}$, and $set_{5}$, which indicate identical matches (21958). Their results support each other so the identity 21958 is finalized as the identity of the given reference star. Because we do not actually know a true identity of an imaged star in a lost-in-space scenario, this verification process can provide redundant opportunities to filter out incorrect matchings with the reference star. In a dynamic condition, this final verification step could be more important than the prior two procedures for improving the robustness of the algorithm.

## 4. Dynamic Environment Analysis

### 4.1. Effects of High Angular Rates

This section begins with simulation test results to show how the slewing motion of the sensor affects the signal-to-noise ratio and centroid accuracy. The simulations were performed to provide two primary values. The first value to be defined is a reasonable threshold of stellar magnitude for on-board database generation. In dynamic conditions, the centroid accuracy varies greatly with the brightness of stars. The shape of the stars become streaks across several pixels and this trail is longer with an increase in the angular rate. The intensity of the stars is distributed among the corresponding pixels, which results in a decrease of the signal-to-noise ratio. This loss of intensity is greater for dim stars in that it causes problems not only recognizing stars, but also in degrading the attitude determined. However, the star tracker does not guarantee availability for the entire sky if we use only bright stars. Moreover, the identification algorithm has difficulty performing correctly with only a small number of bright stars in such dynamic situations. This is a trade-off that requires a threshold appropriate for the brightness of the stars. The second value to be determined is the magnitude of the error that should be considered in the simulations for performance verification. The errors corresponding to the defined brightness threshold could give validity that the algorithm is sufficient to handle the dynamic environment.

To predict the number of photons detected by an image sensor for a given stellar magnitude, intensity models should be defined. The intensity model used in this paper is given by

(29) $$I_{s}=\frac{1}{4}\eta_{Q}t_{e}\pi D^{2}\phi_{0}\cdot 10^{-0.4m},$$

where $\eta_{Q}$ is the quantum efficiency of the image detector, $t_{e}$ is the exposure time, D is the aperture diameter, $\phi_{0}$ the reference irradiance of a zero-magnitude star, and m is the apparent stellar magnitude of an imaged star. Then, the number of photons from the star is given as

(30) $$e_{s}=\frac{\lambda }{hc}\int_{\lambda_{1}}^{\lambda_{2}}\frac{1}{4}\eta_{Q}\left(\lambda \right)t_{e}\pi D^{2}\phi_{0}\left(\lambda \right)\cdot 10^{-0.4m}\;d\lambda ,$$

where λ is the wavelength of incident rays, and h and c are Plank’s constant and the speed of light.

A shape model of stars was also defined to make a star image on the sensor plane. Usually, the starlight goes through a circular aperture and appears as a disk due to diffraction. The disk made by starlight is called an “Airy Disc”, and represents a star in a static condition. The intensity of the star image that forms an airy disk is given by

(31) $$I_{x}=I_{s}{\left[\frac{2J_{1}\left(x\right)}{x}\right]}^{2}.$$

Here, $I_{s}$ is the intensity of a star with the given magnitude and $J_{1}\left(x\right)$ is the Bessel function of the first kind of order unity. If a slewing motion exists, the center of the airy disk will be moved during the exposure time, and the disks on the motion track overlap and depict a smearing effect from the motion. The effect of the motion was depicted by rotation matrix computed by well-known Euler’s rotation theorem. The simulations were performed 10,000 times at each angular rate, and the maximum rate was up to 10 deg/s by referring to the value in [5]. More details about the parameters used in this simulation are listed in Table 4.

**Figure 7:**
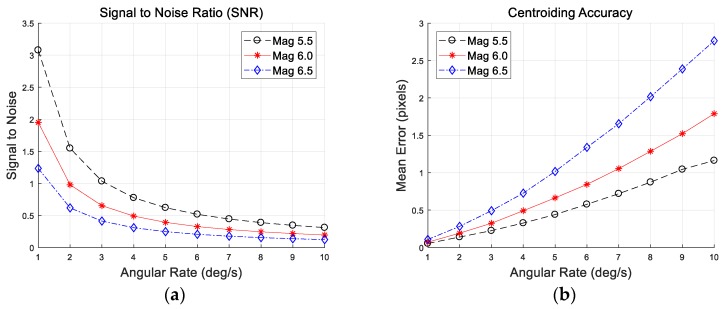
(**a**) Signal-to-noise ratio; (**b**) mean error of a centroid as a function of rates.

Figure 7 shows results of the simulation tests for stars of varying stellar magnitudes. As you can see in the results, the signal-to-noise ratio gradually decreases with increasing angular rate, and leads to a serious drop in the centroid accuracy. For each stellar magnitude, they go through an identical trend with increasing angular rate, but the loss of intensity is more severe with dim stars than with a bright one. The results for a given stellar magnitude at an angular rate of 10 deg/s are listed in Table 5. The stars of 6.5 stellar magnitude have substantially larger error, meaning that the centroid accuracy is 2.76 pixels. This decreased accuracy is unfavorable for subsequent attitude determination, even if the stars are identified correctly.

**Figure 8:**
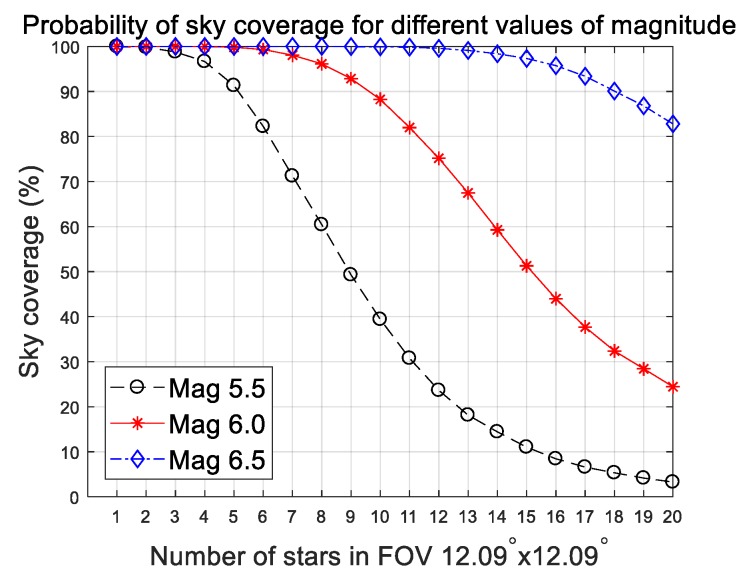
Probability of sky coverage in a 12.09°×12.09° field of view (FOV) for different stellar magnitudes.

To determine the magnitude threshold able to satisfy the desired availability of sky, the probability of the number of stars in the field of view (FOV) should be considered at the same time. The proposed method requires at least six stars, so the criteria for availability were selected based on this value. The Hipparcos stellar catalog was scanned with 6752 uniformly distributed directions over the entire sky to investigate the availability. Strictly speaking, extending the magnitude threshold to dim stars increases availability. Figure 8 shows sky coverage plots and Table 6 summarizes the results at each stellar magnitude. The 5.5 stellar magnitude has a lack of sky coverage that amounts to a probability of only 82.24%. Thus, we concluded from these results that the threshold for stellar magnitude should be 6.0, considering both availability and centroid accuracy. As a result, the proposed algorithm can tolerate centroid error of about 1.79 pixels, which is the worst-case error at a slew rate of 10 deg/s.

### 4.2. On-Board Database Generation

The Hipparcos stellar catalog was used in this work to construct an on-board database of the star tracker. As described in the previous section, the sensitivity of the sensor was assumed to be an apparent magnitude of 6.0 Mv for centroid accuracy and the feasibility of our algorithm over the entire sky. Therefore, a total of 5850 stars are included in the database. Those stars are assumed to be detected by the star tracker with the defined sensor configuration in Table 4. If we want to identify imaged stars in the sky, the on-board database should be configured from the catalog prior to matching the sequences. The database consists of corresponding patterns of catalog stars generated in the same way described in the previous section. The proposed algorithm builds one set of patterns with the nearest four stars around the reference star (see Equation (18)). This is similar to the usage of specific geometry in the first class of algorithms in terms of a fixed number of stars. However, they extract all combinations of the geometry. This could be the major drawback of the first class of algorithms, resulting in waste of computational time and memory. Their logic in searching for all pairs or geometries of stars has proven to be more inappropriate for finding the best candidate than a pattern-matching strategy. Because simplicity of the invariant alone cannot effectively handle the dynamic environment, increased attempts by all pairs or geometries do not guarantee a performance enhancement.

**Figure 9:**
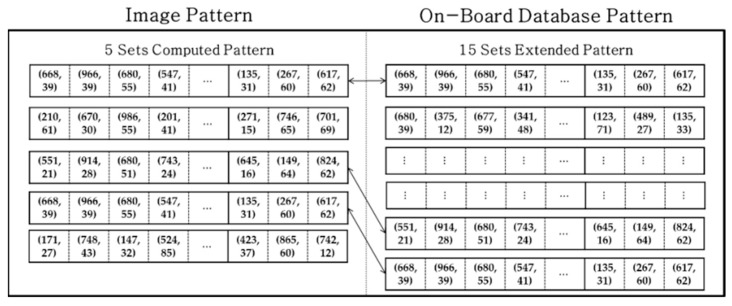
Image and extended database grid pattern for one reference star.

To resolve issues such as those discussed above, our strategy for establishing a database was to use only the six nearest stars to form the database. The one more selected star takes into account possible contingencies in which position errors could change the order of the nearest stars. If we draw four stars from the nearest six stars, it is equivalent to a combination problem of four out of six (=C46). This may result in 15 sets of five stars for the one reference star. Afterward, procedures identical to those described in the previous section are applied to each set. As one can see in the Figure 9, the extended pattern composed of 15 sets of discretized grid patterns was stored in the database for every star cataloged.

## 5. Simulation Study

### 5.1. Setup for Simulation

The performance of the proposed algorithm was demonstrated using images synthesized under several situations. The synthesized star images were created by scanning the sky from the Hipparcos stellar catalog with 6752 uniformly distributed bore-sight directions covering the entire celestial sphere. Two noise sources were considered: Positional and false star noise. The positional noise was assumed to be random Gaussian noise with its noise level defined with standard deviations. The noise was added to both x and y coordinates of true centroid location of each imaged star and the standard deviation of the noise was increased from 0 to 150 arc-seconds. The range of the positional noise was determined from previous simulation results whereby 150 arc-seconds were the same as 1.77 pixels in the defined sensor specification. Moreover, the star image could contain several false stars caused by image spikes, hot spots, or by other objects in space. The false stars were projected on the sensor plane at random locations within the FOV, and their number ranged from 1 to 10. All the methods, including the proposed and benchmarked ones, were implemented using the same platform for fair comparison of time performance. They were accessed using MATLAB R2017a software run on an Intel Core I5 processor (3.4 GHz, 8 GB RAM).

### 5.2. Case 1: Single Identification Process for One Reference Star

**Figure 10:**
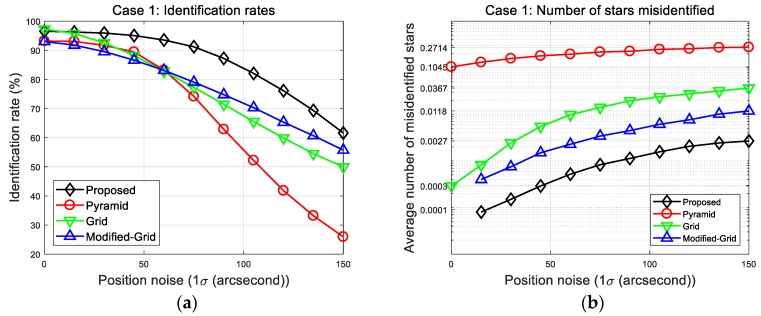
Case 1: (**a**) Identification rates; (**b**) number of misidentified stars.

To analyze the performance of the algorithm, simulations were carried out for three different cases. The first case was to measure the fundamental performance of each algorithm as it performed star identification for a single reference star. In this case, the identification rates and average number of misidentified stars were provided to allow comparison of the proposed algorithm with others. The positional error was introduced as a single noise source via random Gaussian noise with 1σ ranging from 0 to 150 arc-seconds. The simulation was performed 10,000 times at each noise level. Figure 10 shows plots of the identification rates and number of misidentified stars.

As shown in the first result plots, the new algorithm features identification rates far superior to those of other algorithms with respect to position error. Despite not using all the surrounding stars, the new algorithm provides more reliable identification rates, and it also shows the least number of misidentified stars at all noise levels. This result is attributed to the use of more complicated patterns. These become more robust against position error by appropriately giving one more degree of freedom to the cell index. The results at 150 arc-seconds position error are listed in Table 7 to facilitate comparison of the algorithms. It is noteworthy that the identification speed of the proposed methods is much faster without the need for the look-up table technique. The proposed method takes about 1.35 ms for a single identification process, which is similar to grid and modified grid algorithms. These results are achieved by efficiently reducing the number of candidates in the initial matching stage so that only a small part of the database is searched. Our database consists of 5850 stars and 87,330 associated patterns, so that 87,330 candidates exist prior to the initial step. After the initial match, the average number of remaining candidates in the database is only 147.8—A significantly reduced number compared with the size of the original database. Thus, one can conclude from the simulation results that the proposed method could provide more favorable performance for this error type in terms of identification rates, as well as providing the lowest erroneous matching.

### 5.3. Case 2: Identification of Several Reference Stars

**Figure 11:**
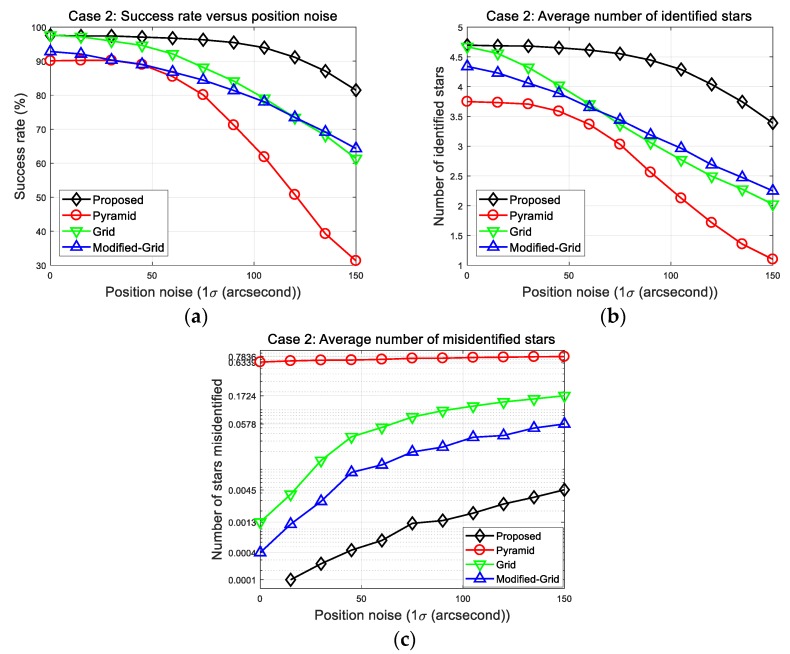
Case 2: (**a**) Success rates; (**b**) number of identified stars; (**c**) number of misidentified stars.

In this section, we address the performance of each algorithm in the case of multiple reference stars. Case 2 simulations were performed under the same conditions as those in Case 1, except that several reference stars were used. This is more consistent with the actual situation because it is barely possible to identify only one imaged star. The identification procedures were performed for five stars in one image, and it was considered successful only if at least two of them were correctly recognized to make the subsequent attitude determination. The results are shown in Figure 11.

The success rates of all methods were improved due to the additional opportunities of the new identification procedures. Moreover, it is obvious that the grid and modified grid algorithm performed better than the pyramid algorithm did under the given position error. This means that the application of a pattern matching strategy is essential to acquire robustness, which could not be provided using invariants alone. The resultant figures also show that both success rates and number of correctly identified stars of the other benchmark algorithms decrease more rapidly than the new algorithm as the position error increases. The resulting parameters are listed in Table 8. The success rate of the proposed algorithm is 81.4% at 150 arc-seconds position error, which is superior to other algorithms, along with the least number of misidentified stars. This result was caused by the last verification procedure, which increased reliability with respect to the erroneous matching of stars.

### 5.4. Study Parameters for the Proposed Method

**Figure 12:**
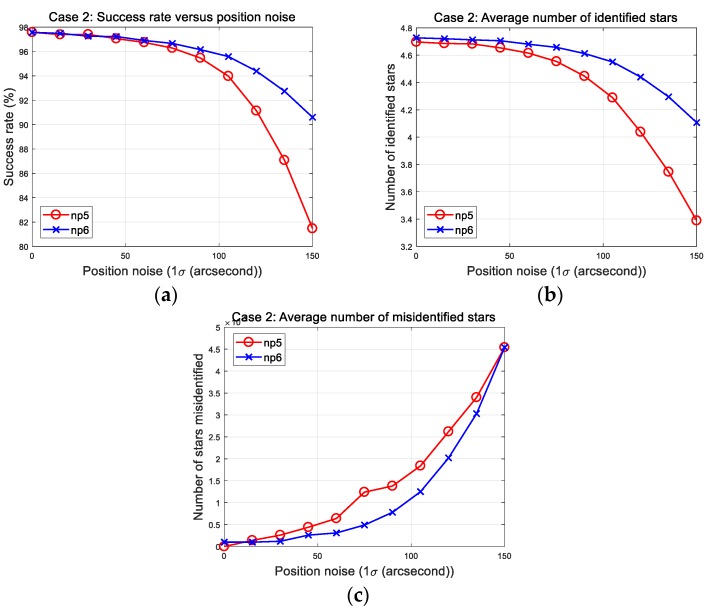
Case 2: Parameter study of patterns using the nearest five and six stars. (**a**) Success rates; (**b**) number of identified stars; (**c**) number of misidentified stars.

The proposed algorithm creates a database that includes the six nearest stars. This is one of several redundant strategies to enhance the capability of the algorithm because the pattern of the reference star is generated using the five nearest stars. However, the image pattern was also modified to be generated using the six nearest stars as with the database strategy. Then, the five sets of computed patterns (Figure 9) were expanded to produce 15 sets of an extended pattern. This modification is examined in the Case 2 simulations. The results are shown in Figure 12, in which np5 and np6 mean the case using the nearest five and six stars, respectively.

In the result plots, success rate and average number of identified stars are clearly improved by using the extended pattern from the six nearest stars. The resulting parameters at 150 arc-seconds are listed in Table 9. Although the running time of the algorithm increased from 16.54 to 24.91 ms, the important thing is that it maintained the same level of misidentification. Thus, the algorithm can provide enhanced performance when the reference star pattern is created utilizing the nearest six stars.

### 5.5. Case 3: Overall Performance for the Entire Image

The Case 3 simulations compare the overall performance of the algorithms for the entire image when a false star exists with a position error. Unlike in the previous section, the simulation performed the identification procedure for all the stars imaged to measure the overall performance, while the position error was fixed at 150 arc-seconds. In the Case 3 simulations, the pyramid algorithm was excluded due to its lack of robustness, and the proposed algorithm used star identification with patterns produced using the both five and six nearest stars for performance comparison. Another desirable ability of a star identification algorithm is reliable performance regardless of the FOV size. This was tested in this case by changing the camera to achieve narrow and wide FOVs. In the case of a wide FOV, the image resolution changed to 1024 × 1024 pixels (twice that of a narrow case), so that the FOV was enlarged from 12.09° to 23.98°.

In contrast with the previous simulations, the false stars appeared at random locations within the FOV and the number of the false stars was increased from 1 to 10 in both cases. In addition, the false stars were assumed to be false negative error so that they did not exist in the database. All the simulations were performed 10,000 times for each false star number. The average run time, number of correctly matched stars, and number of misidentified stars are presented to demonstrate the effectiveness of the proposed algorithm in more complicated situations. The memory usage of all methods is compared at the end of this section.

**Figure 13:**
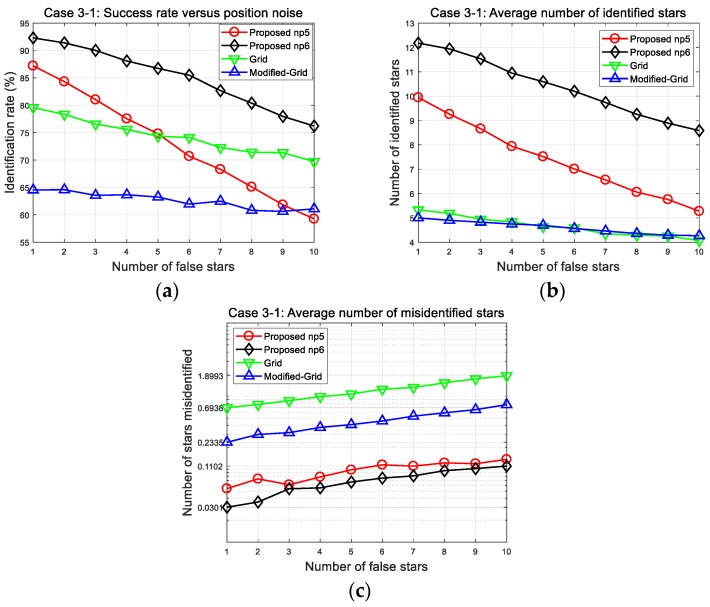
Case 3-1: (**a**) Success rates; (**b**) number of identified stars; (**c**) number of misidentified stars.

First presented are the results of the narrow FOV case (Case 3-1 in Figure 13). The success rates of all algorithms tend to decrease with increase in the number of false stars. These results are incurred by the probability that the spurious stars are likely to generate patterns entirely different from the stored one. In dynamic situations, this error factor is inevitable, but the proposed method can handle such cases due to its pattern complexity and matching strategy.

The resultant parameters for the presence of 10 false stars and 150 arc-seconds position error are summarized in Table 10. The average number of stars is about 26.4 in the narrow FOV including 10 false stars. When we consider that the number of true stars is about 16.4 compared with 10 false stars, this would be a very challenging condition under which to identify stars. One can observe that the proposed algorithm produces a remarkable performance in the number of correctly identified stars. Furthermore, it should be noted that a failure to match is preferred to the mismatching of stars in the identification process, regarding later navigation. This makes obvious that this method is more appropriate for dynamic conditions because the number of misidentified stars remains at the lowest level, even with increase in the number of false stars.

**Figure 14:**
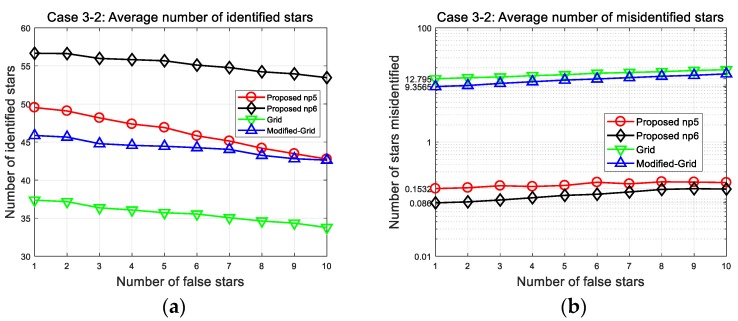
Case 3-2: (**a**) Number of correctly identified stars; (**b**) number of misidentified stars.

The second results are of the wide FOV case (Case 3-2 in Figure 14). The average number of stars in one image is increased significantly (relative to the narrow FOV). As more stars are included to be identified, greater differences in the number of misidentified stars and identification time results. The results from the comparison based on 150 arc-seconds position error with 10 false stars are presented in Table 11.

When the FOV contains about 73 stars, the identification time of the proposed algorithm is 0.18 s (np5) and 0.40 s (np6), which is dramatically shorter than the 2.89 s of the grid algorithm, 5.26 s of the modified grid algorithm, respectively. These results imply the advantage of patterns using fixed number of stars: It makes the identification time almost linearly proportional to the number of stars. We can see that the identification time of the grid and modified grid algorithm increases more steeply in the wide FOV case because their patterns are based on the surrounding stars. From the results, we can recognize that proposed initial matching scheme is more efficient than a look-up table generation in other pattern matching algorithms. Thus, the proposed method could make the algorithm more reliable for high-rate applications, with faster run-time as well as robust performance.

In this study, a performance indicator is also proposed to facilitate the comparison of each algorithm. The performance indicator is defined by taking into account a subsequent attitude determination and navigation purpose. It is because a larger number of correctly matched stars directly affect the accuracy and reliability of systems, that one can consider the ratio between the average numbers of correctly and incorrectly matched stars. In addition, the average number of stars in the FOV could be slightly different due to arbitrary bore sight directions, so the ratio is divided by this value. Here, the performance indicator can be given as

(32) $$P_{index}=\frac{Avg.\;\#\;of\;correctly\;identified\;stars}{Avg.\;\#\;of\;misidentified\;stars}\times \left(\frac{Success\;rates}{Avg.\;\#\;of\;stars}\right).$$

**Figure 15:**
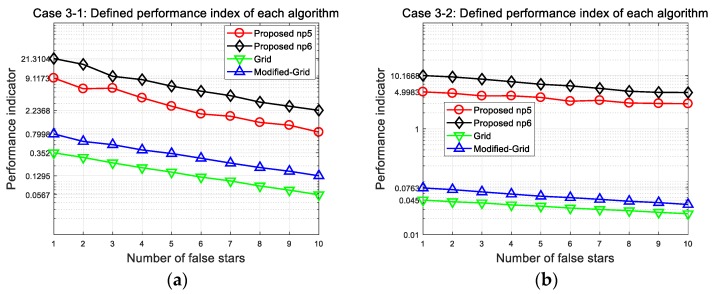
Performance index of each algorithm at 150 arc-seconds position error: (**a**) Case 3-1 indicates performance with narrow FOV; (**b**) Case 3-2 indicates performance with wide FOV.

The defined performance indicator of each algorithm is shown in Figure 15 and summarized in Table 12. As one can see in the plots, the proposed method exhibits the best performance for both narrow and wide FOV, and the difference grows more distinct for the wide FOV. Although the fixed number of stars is used for the pattern generation, the proposed method can preserve the least mismatched star numbers to provide additional opportunities for star identification trials. This could be one significant contribution of our algorithm, that a highly reliable navigation system could be developed by minimizing the adverse effect of incorrectly matched stars.

Memory usage is also an important parameter for on-board algorithm implementation. The memory usage of all methods is summarized in Table 13. The proposed method requires 2275 and 2347 KB for the databases of Case 3-1 and 3-2, which are larger than those required for the grid and modified grid algorithms. This is because the proposed algorithm stores the extended fifteen sets of patterns for each reference star. However, the bigger database size is necessary to provide a feasible solution in a dynamic situation. One more issue regarding memory usage is that the memory usage of the proposed algorithm will not increase with the FOV because its result relies upon a fixed number of stars to construct the pattern. Only the grid and modified grid algorithms showed an increase in their memory usage for wide FOVs.

## 6. Conclusions

A novel star identification algorithm for use in lost-in-space scenarios was addressed along with simulation studies to support the proposed process. It was intended to resolve the deficiencies of previous methods by combining the advantages of each strategy. Instead of using star coordinates in the pattern generation, it uses singular values to construct a pattern. This strategy turns out to provide improved robustness (against not only positional error, but also against false star noise) with a sequence of three effective matching schemes. The proposed algorithm was verified in depth by exhaustive simulation. The results imply that the new idea provides a highly reliable performance even with a wide range of noise sources, compared to those of the other benchmarked algorithms. In addition, the new process outperforms others when considering various numbers of stars. It can be applied from narrow to wide sensor FOVs, which ensures a more rapid identification speed as the patterns are exploited from a specific number of selected stars, not all the surrounding stars. The results of this study could be beneficial for improving accuracy and reliability of existing star sensor systems for more rapid and more stable attitude determination in lost-in-space situations.

## Figures and Tables

**Table 1:** Defined discretization parameter and distribution range of each subset.

Parameter for Pattern	1st Singular Value	2nd Singular Value	3rd Singular Value
Step size	$1\times 10^{-4}$	$15\times 10^{-4}$	$15\times 10^{-4}$
Distribution range subsets of 3 stars	$\left[\begin{array}{cc} 1.7275, & 1.7325 \end{array}\right]$	$\left[\begin{array}{cc} 0, & 0.1 \end{array}\right]$	$\left[\begin{array}{cc} 0, & 0.05 \end{array}\right]$
Distribution range subsets of 4 stars	$\left[\begin{array}{cc} 1.995, & 2.0 \end{array}\right]$	$\left[\begin{array}{cc} 0, & 0.12 \end{array}\right]$	$\left[\begin{array}{cc} 0, & 0.1 \end{array}\right]$
Distribution range subsets of 5 stars	$\left[\begin{array}{cc} 2.23, & 2.238 \end{array}\right]$	$\left[\begin{array}{cc} 0, & 0.15 \end{array}\right]$	$\left[\begin{array}{cc} 0, & 0.1 \end{array}\right]$

**Table 2:** Example of the initial matching in the case of threshold 1.

Threshold: 1	Pattern from the Subsets of Five Stars	
Reference star	2755	64
Search range of database	2754, 2755, 2756	63, 64, 65

**Table 3:** Example of results after the second procedure for one reference star.

	Number of Votes	Matched ID	True ID
**$Set_{1}$**	8	21958	21958
**$Set_{2}$**	7	21393	21958
**$Set_{3}$**	10	21958	21958
**$Set_{4}$**	4	−1	21958
**$Set_{5}$**	9	21958	21958
**Final results**	21958		

**Table 4:** Simulation parameters for a dynamic condition.

**Field of view**	12.09 × 12.09 degrees
**Resolution**	512 × 512 pixels
**Angular resolution**	0.0236 degree
**Pixel size**	20 × 20 μm
**Diameter**	60 mm
**Exposure time**	100 ms
**Response non-uniformity**	3% of mean
**Dark current noise**	200 $e^{-}/\mathrm{pixel}/s$
**Dark signal non-uniformity**	80 $e^{-}/\mathrm{pixel}/s$
**Background noise**	20 Mv stellar magnitude

**Table 5:** Centroid accuracy and signal-to-noise ratio at 10 deg/s angular rate.

Magnitude Limit	5.5 Mv	6.0 Mv	6.5 Mv
Centroid mean error (pixels)	1.1630	1.7893	2.7635
Signal to noise ratio	0.3112	0.1963	0.1239

**Table 6:** Probability of availability of at least six stars.

Magnitude Limit	5.5 Mv	6.0 Mv	6.5 Mv
Probability	82.2423%	99.3187%	100%

**Table 7:** Case 1: Comparing results of star identification methods at 150 arc-seconds position error.

Algorithm	Proposed	Pyramid	Grid	Modified Grid
Identification rate	61.60%	25.93%	49.94%	55.73%
Average number of misidentified stars	0.0027	0.2761	0.0367	0.0118
Average elapsed time for one identification	1.3545 ms	71.6264 ms	1.7440 ms	2.5140 ms

**Table 8:** Case 2: Comparing results of star identification methods at 150 arc-seconds position error.

Algorithm	Proposed	Pyramid	Grid	Modified-Grid
Success rate	81.47%	31.32%	61.36%	64.31%
Average number of correctly identified stars	3.3888	1.1006	2.0260	2.2495
Average number of misidentified stars	0.0045	0.7836	0.1724	0.0578
Average elapsed time for whole image	16.5382 ms	335.3134 ms	8.6729 ms	11.9913 ms

**Table 9:** Case 2: Parameter study of proposed methods at 150 arc-seconds position error.

Algorithm	5 Nearest Stars	6 Nearest Stars
Success rates	81.47%	90.60%
Average number of correctly identified stars	3.3888	4.1063
Average number of misidentified stars	0.0045	0.0024
Average elapsed time for whole image	16.5382 ms	24.9145 ms

**Table 10:** Case 3-1: Comparing results of star identification methods in 12.09°×12.09° FOV.

Algorithm	Proposed (np5)	Proposed (np6)	Grid	Modified-Grid
Average number of stars in one image	26.5297	26.5365	26.3358	26.3901
Success rates	59.27%	76.21%	69.72%	61.10%
Average number of correctly identified stars	5.2739	8.5832	4.0657	4.2685
Average number of misidentified stars	0.1360	0.1102	1.8993	0.7633
Average elapsed time for whole image	0.0512 s	0.1461 s	0.0398 s	0.0513 s

**Table 11:** Case 3-2: Comparing results of star identification methods in 23.98°×23.98° FOV.

Algorithm	Proposed (np5)	Proposed (np6)	Grid	Modified-Grid
Average number of stars in one image	73.3752	73.3345	73.7020	73.9442
Success rates	100%	100%	100%	100%
Average number of correctly identified star	42.7619	53.4565	33.7710	42.6252
Average number of misidentified star	0.1953	0.1502	18.4740	15.6191
Average elapsed time for whole image	0.1805 s	0.3999 s	2.8926 s	5.2578 s

**Table 12:** Defined performance index at 150 arc-seconds position error with 10 false stars.

Algorithm	Proposed (np5)	Proposed (np6)	Grid	Modified-Grid
Case 3-1: NFOV	0.8664	2.2368	0.0567	0.1295
Case 3-2: WFOV	2.9837	4.8531	0.0248	0.0371

**Table 13:** Summary of average memory requirements for narrow and wide FOVs.

Algorithm	Proposed	Grid	Modified-Grid
Memory usage—NFOV	2275 KB	341 KB	326 KB
Memory usage—WFOV	2347 KB	1164 KB	1134 KB
